# Future in Pediatrics: prevention in maternal child chronic diseases in the first 1000 days

**DOI:** 10.1093/eurpub/ckac129.672

**Published:** 2022-10-25

**Authors:** B Hugonin-Rao

**Affiliations:** Maternal Newborn Child Health, Azienda USL Toscana Centro, Florence, Italy

## Abstract

**Introduction:**

The screening and management of obesity, metabolic chronic conditions conditions and genetic predisposition, before and during pregnancy, improve the effects of therapies and reduce the rate congenital diseases, metabolic fetal disorders and early chronic diseases during first 1000days. The maternal child health promotion program ‘Future in Pediatrics’ is dedicated to women in preconception and in early pregnancy, with three steps: 1) a specific survey, 2) a personalisated plan 3) the management of chronic conditions (diabetes, hypertension, overweight, metabolic disorders) and surveillance during the first maternal child 1000 days. Encouraged results

**Methods:**

In 2019-2021, FUTURA project involved 460 women (before,during and after pregnancy) into 2 groups: group 1 of 280 women, with overweight/ obesity, metabolic conditions, malnutrition, and hypovitaminosis, group 2 of 180 women without chronic conditions, but physical inactivity, high level of homocysteine (60 to 100 μmol/L) and with predisposition for cardiovascular diseases. We have involved the patients in a survey of 100 questions about health, lifestyle, habits, sleep and in a daily diary. In the second time we have realized a personalised and educational program for diet, supplementations sleep, physical activities, health routine.

**Results:**

The 2 groups of women improved the quality of their health and the management of weight, chronic and metabolic conditions, with impact on reproductive and perinatal health, reduction of inflammatory status and metabolic parameters.We have observed a great influence on cardiovascular health in both groups and decreased of homocysteine levels in the 2nd group. During first 1000 days 393 women, involved into the program, continued with the healthy and preventive routine with zero cases of weight and metabolic chronic disorder in their children.

**Conclusions:**

This research that the prevention in perinatal health influences the children's health.

**Key messages:**

• The origins of chronic and metabolic conditions are in uterine life before the conception. The unhealthy lifestyle influenced fertility, cardiovascular health and child health.

• The cardiovascular health and metabolic chronic conditions are influenced by first 1000 days health and habits.

